# Value of black blood T2* cardiovascular magnetic resonance

**DOI:** 10.1186/1532-429X-13-21

**Published:** 2011-03-14

**Authors:** Gillian C Smith, John Paul Carpenter, Taigang He, Mohammed H Alam, David N Firmin, Dudley J Pennell

**Affiliations:** 1 CMR Unit, Royal Brompton Hospital, Sydney Street, London SW3 6NP, UK; 2 National Heart & Lung Institute, Imperial College London, Guy Scadding Building, Dovehouse Street London SW3 6LY, UK

## Abstract

**Purpose:**

To assess whether black blood T2* cardiovascular magnetic resonance is superior to conventional white blood imaging of cardiac iron in patients with thalassaemia major (TM).

**Materials and methods:**

We performed both conventional white blood and black blood T2* CMR sequences in 100 TM patients to determine intra and inter-observer variability and presence of artefacts. In 23 patients, 2 separate studies of both techniques were performed to assess interstudy reproducibility.

**Results:**

Cardiac T2* values ranged from 4.5 to 43.8 ms. The mean T2* values were not different between black blood and white blood acquisitions (20.5 vs 21.6 ms, p = 0.26). Compared with the conventional white blood diastolic acquisition, the coefficient of variance of the black blood CMR technique was superior for intra-observer reproducibility (1.47% vs 4.23%, p < 0.001), inter-observer reproducibility (2.54% vs 4.50%, p < 0.001) and inter-study reproducibility (4.07% vs 8.42%, p = 0.001). Assessment of artefacts showed a superior score for black blood vs white blood scans (4.57 vs 4.25; p < 0.001).

**Conclusions:**

Black blood T2* CMR has superior reproducibility and reduced imaging artefacts for the assessment of cardiac iron, in comparison with the conventional white blood technique, which make it the preferred technique for clinical practice.

## Introduction

Beta thalassaemia major (TM) is an inherited anaemia and without regular blood transfusions patients die during childhood. Although transfusions improve health and survival, the consequent tissue iron deposition leads to organ damage in the long term. Those treated only with transfusions usually die early from cardiac failure secondary to myocardial siderosis [1,2], and therefore long term iron chelation treatment with deferoxamine became the standard treatment of choice to control iron levels from its introduction in the late 1960s. However, long term use of deferoxamine often does not prevent the eventual accumulation of myocardial iron, left ventricular dysfunction and death from heart failure. Although myocardial dysfunction is directly linked with cardiac iron burden [3], it often occurs late and can be hard to reverse once established. Therefore direct assessment and early treatment of iron loading is pivotal in the management of these patients, along with tailored cardiac chelation therapy which may reverse or prevent myocardial dysfunction [4,5]. Over the last decade, there has been a marked reduction in deaths attributable to tissue iron overload within the UK which coincides with the introduction of T2* cardiovascular magnetic resonance (CMR) as a diagnostic tool [6]. Life expectancy for patients in high income countries with good compliance to therapy is now over 50 years, although survival in low income areas is still poor [7].

The relaxation parameter T2* is shortened by field inhomogeneities induced by tissue iron. Gradient echo CMR can measure T2* decay, and a value of <20 ms is considered to be indicative of iron overload [8]. The first T2* sequence described was a multi breath-hold mid-ventricular short axis acquisition with end-diastolic gating [8]. A single breath-hold sequence was later developed which reduced scan time, improved image registration between images, and had good reproducibility [9]. This technique has become the mainstay of clinical evaluation and follow-up of TM patients. It has been installed on CMR scanners from different vendors at multiple sites throughout the world with reproducible results [10], and has been used to investigate the cardiac efficacy of chelating agents [11-13]. Despite this success, the white blood technique has some undesirable characteristics: the contrast between blood pool and myocardium may be suboptimal, and despite flow compensation, artefacts from motion and blood flow may compromise measurement accuracy. More recently, a double inversion recovery 'black blood' sequence has been reported which suppresses the blood signal, and a preliminary comparison with white blood T2* imaging suggested good reproducibility [14]. The aim of this study was therefore to compare the inter and intra-observer reproducibility between the established white blood sequence and the newer black blood sequence in a large group of patients, and to evaluate the interstudy reproducibility and artefacts when using the black blood sequence.

## Materials and methods

We studied 100 TM patients to compare the black blood and white blood acquisitions. The cohort included 50 consecutive patients with cardiac siderosis (T2* <20 ms) and 50 consecutive patients with no cardiac iron (T2* > 20 ms) as measured using the conventional white blood T2* acquisition. Cardiac T2* values ranged from 4.5 to 43.8 ms across all patients. The age range was 11-50 years (mean 27) and 51% were female. All patients underwent both the conventional white blood and the new black blood T2* scan to test intra and inter-observer reproducibility. To test inter-study reproducibility, 23 patients with T2* values from 6.9 to 46.1 ms had a second scan for both black and white blood acquisitions on the same day.

All patients were scanned using a 1.5 T scanner (Siemens Sonata, Erlangen, Germany) with a 6 channel phased array cardiac receiver coil and ECG gating. A mid-ventricular short axis slice was obtained for all scans. The white blood acquisition used a multiecho gradient-echo sequence (flip angle 20°, matrix 128 × 256 pixels, sample bandwidth 810 Hz/pixel, slice thickness 10 mm and field of view 40 cm). The short axis images were acquired within a single breath-hold at 8 echo times from 2.54 to 17.90 ms at approximately 2 ms increments. For white blood diastolic imaging, the images were acquired immediately after the R-wave. For the black blood acquisitions, a double inversion recovery (DIR) pulse was applied on the R-wave and the inversion time extended into diastole, generating a similar set of 8 images at increasing echo times [14].

**Figure 1:**
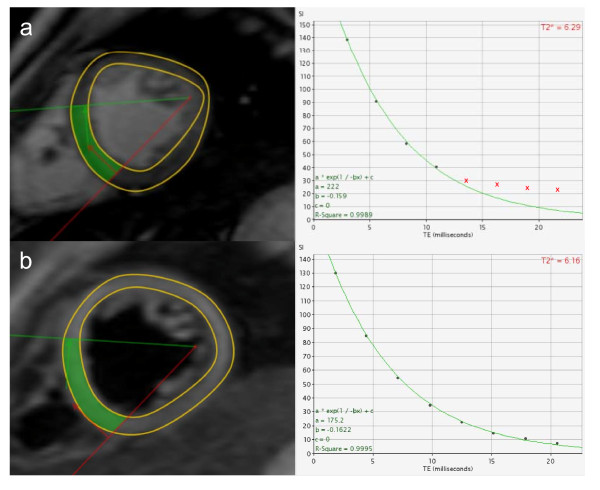
**Signal decay is rapid in heavily iron loaded hearts causing the curve to plateau for the longer echo times**. The upper frame shows how later points that fall below background noise level are removed to improve curve fit (red crosses) using the truncation method (R^2^= 0.9989 with truncation). For the black blood acquisition (b) background noise is reduced thus the curve fit is good for the full range of echo times without truncation (R^2 ^= 0.9995) which reduces the likelihood of analysis errors.

Image analysis was performed using Thalassaemia tools (a plug in of CMRtools, Cardiovascular Imaging Solutions, London, UK). A full thickness ROI incorporating the ventricular septum was selected avoiding blood pool and cardiac vessels [15,16]. T2* was derived by using an exponential curve fit, SI = SI_0_.*exp*^-(*TE*/*T*2*)^, where SI is signal intensity, TE is the echo time and SI_0 _is a constant representing signal intensity at time zero. A truncation method was used to account for background noise at the longer echo times and improve curve fit[17], although this was not generally necessary for the black blood acquisitions (Figure 1). Analyses for all sequences were performed by two experienced investigators. Each data-set was anonymised and presented to observers in random order. Intra-observer reproducibility was assessed using 2 sets of measurements derived by observer 1. To test inter-observer reproducibility, the first read results were compared with those of observer 2. Inter-study reproducibility was assessed by observer 1 using data from the repeated studies. Analysis of image quality was conducted by 2 observers using the following 5 point scale: 0 - Very poor image quality with unusable images; 1 - Poor image quality, just able to make out the heart but a lot of artifact; 2 - Average image quality, not all of septum clearly seen and a lot of artifact; 3 - Good image quality with moderate septal artifact; 4 - Very good quality, with minimal septal artifacts; 5 - Excellent image quality with no significant septal artifact. To quantify the reproducibility, the coefficient of variance (CoV) was calculated from the standard deviation of the differences between analyses divided by their mean. A paired, two-tailed Student's t-test was performed on the natural log transformation of squared differences between methods to assess differences in measurement variability between methods [18]. Friedman's test was used to analyze the image quality scores. A *p *value of < 0.05 was considered significant. The local research ethics committee approved the study and patients gave informed consent.

## Results

**Figure 2:**
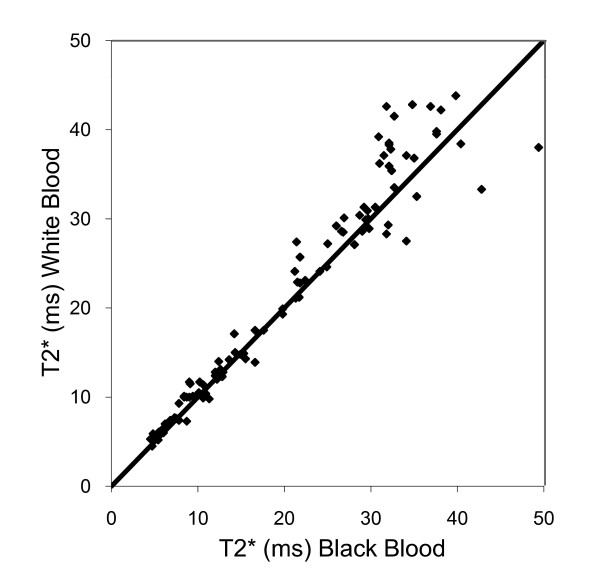
Scatter plot of myocardial T2* values obtained from white blood and black blood acquisitions, showing the line of identity. Agreement is good for patients with T2* ≤ 20 (iron overloaded) but discrepancy increases with increasing T2*.

There was no significant difference between the mean cardiac T2* values for all 100 patients for the black blood acquisition and the conventional white blood sequence with diastolic imaging (20.5 ms vs 21.6 ms; p = 0.26). Differences between black and white blood acquisitions were also not significant for the iron loaded (n = 50, p = 0.31) and non-iron loaded (n = 50, p = 0.081) subgroups. The scatter plot of white blood vs black blood T2* showed the values lay close to the line of identity (Figure 2).

### Intra and inter-observer reproducibility

**Figure 3:**
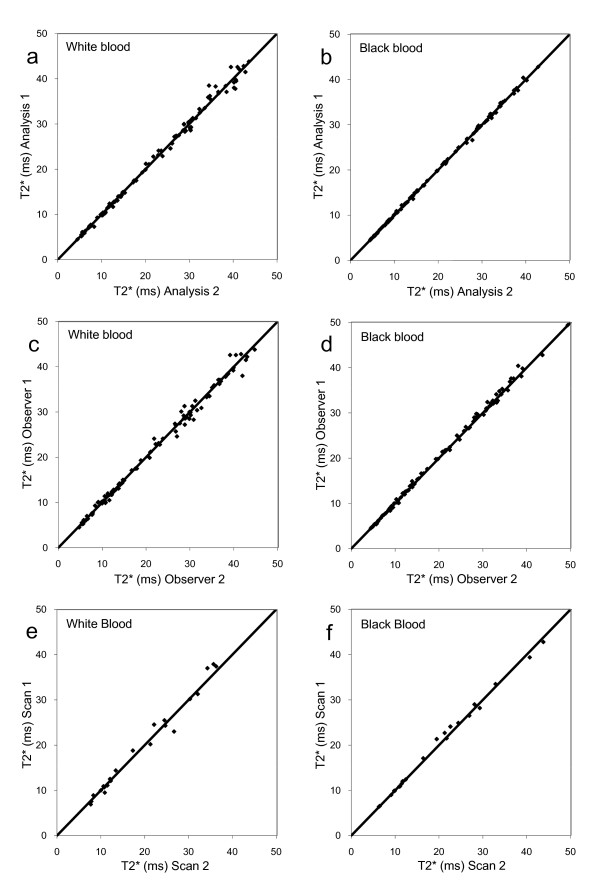
**Scatterplots of reproducibility data, in all cases showing the line of identity**. a) White blood acquisition with scatter plots showing blinded intra-observer reproducibility for myocardial T2* assessment. b) Black blood data showing improved agreement between analyses. c) White blood acquisition with scatter plots showing blinded inter-observer reproducibility for myocardial T2* assessment. d) Black blood data showing improved agreement between analyses. e) White blood acquisition with scatter plots showing blinded inter-study reproducibility for myocardial T2* assessment. f) Black blood data showing improved agreement between analyses.

The intra-observer coefficient of variability (CoV) across the full range of T2* values (n = 100) was significantly lower using the black blood compared with the white blood sequence (1.47% vs 4.23%, p < 0.001). In patients with iron overload (T2* <20 ms) the CoV was significantly lower for black blood than for white blood acquisition (1.68% vs 2.81%, p < 0.001). In patients with no iron overload (T2* >20 ms), the CoV was also significantly lower for black blood than white blood acquisition (1.28% vs 3.88%, p < 0.001). (Figure 3a, b). The inter-observer agreement findings were similarly superior for the black blood acquisition, with a CoV of 2.54% (black blood) compared with 4.50% (white blood) for all patients (p < 0.001), 2.91% vs. 4.28% for iron overloaded patients (p = 0.003) and 2.16% vs. 4.02% for patients with no iron loading (p = 0.03) (Figure 3c, d)

### Inter-study reproducibility

For all patients (n = 23), the CoV for inter-study reproducibility was superior for black blood compared with white blood acquisition (4.07% vs 8.42%, p = 0.001). (Figure 3e, f) In patients with a T2* <20 ms (n = 12) the CoV was 2.21% for black blood and 7.04% for white blood (p = 0.003). In the patients with no iron loading (n = 11), the CoV was 3.93% and 7.87% respectively (p = 0.15).

### Artefact scoring

The mean artefact score was superior for the black blood acquisition compared with white blood (4.57 vs 4.25, p < 0.001).

## Discussion

In this study, we have shown improvement in intra and inter-observer variability together with inter-study reproducibility for black blood T2* imaging over the conventional white blood sequence in a large patient sample. For the conventional white blood sequence with no trigger delay, low signal contrast and the consequent difficulty in defining the myocardial/blood boundary may cause error in T2* measurement. This may result in white signal from the blood pool being incorporated into the decay curve for analysis. The errors may also be exacerbated by mis-registered, high signal blood pool artefacts which can be superimposed on the ROI. Adding the double inversion recovery pulse to generate the black blood images removes most of the blood signal (and hence artefacts) from the myocardium, making the measurement of T2* more robust. Importantly, the greatest disparity between white blood and black blood T2* values was seen when testing inter-study reproducibility in iron overloaded patients where we found a three-fold difference in the coefficient of variation. This discrepancy may be due to different levels of artefact in the myocardial septum resulting in changes in the overall signal intensity in the region of interest between scans. Indeed analysis of artefact scoring showed significantly poorer artefact scoring for the white blood acquisition which could potentially impart significant errors in longitudinal evaluation.

The improved analysis with reduced requirement for truncation of data points in patients with short T2* values (Figure 1), is an additional advantage in reducing analysis errors, particularly in less experienced centres. These findings combined with the improved reproducibility, suggest that the black blood technique should be used for routine clinical practice, providing centres can install a robust sequence as implemented by the scanner manufacturers. There are some disadvantages to the black blood acquisition however. Because of the time taken to achieve blood signal nulling, it is usually necessary to gate on alternate cardiac cycles, unless the RR interval is very long.

## Conclusions

In conclusion, this study shows improved reproducibility and reduced artefacts for the black blood sequence in comparison with the previously validated white blood sequence, and therefore the black blood sequence should be considered the imaging technique of choice.

## Competing interests

The authors declare that they have no competing interests.

## Authors' contributions

GCS carried out data acquisition and interpretation, statistical analysis, manuscript drafting and participated in study design. JPC and MHA performed additional data analysis. TH and DNF designed and optimised the CMR acquisition protocol. DJP was responsible for the initial study concept and the final manuscript. All authors read and approved the final manuscript.

This work was supported by the NIHR Cardiovascular Biomedical Research Unit of Royal Brompton Hospital and Imperial College. Dr He and Dr Alam are currently supported by the British Heart Foundation.
